# Gangrenous Ovarian Cyst Torsion During Pregnancy: A Rare Case Report

**DOI:** 10.7759/cureus.48406

**Published:** 2023-11-06

**Authors:** Prishita Gupta, Abhay Gaidhane, Aditi V Rokade

**Affiliations:** 1 Community Medicine, Jawaharlal Nehru Medical College, Datta Meghe Institute of Higher Education and Research, Wardha, IND

**Keywords:** twist, laparoscopy, acute abdomen, hemorrhage, salpingo-oophorectomy

## Abstract

Torsion of the ovarian cyst is a gynecological emergency that can arise during pregnancy. Regardless of gestational age, surgical procedures should be explored in the course of the growth of adnexal torsion. Although ovarian torsion is not as common during pregnancy and may be an incidental finding, it is always better to stay cautious. If neglected, they may be deemed hazardous to both the baby and the mother. Here we are reporting a case of a 25-year-old multigravida woman who is in the first trimester of her pregnancy, and presented with the complaint of acute abdominal pain for 3 days. On ultrasound, torsion of the right ovarian cyst was found. She underwent laparoscopy and a right-sided salpingo-oophorectomy was done. The biopsy report revealed a benign hemorrhagic cyst. She has been under observation and is asymptomatic, with clear imaging and laboratory values.

## Introduction

The uterus is surrounded by a network of structures called the adnexa. The adnexa involves the fallopian tubes and the ovaries. There is one ovary on each side of the uterus. The ovaries are held in place by a special ligament called the utero ovarian ligament. Thousands of eggs are found in each ovary. Hormones produced in the pituitary gland and the ovary cause one egg to mature and be released each month. This is known as ovulation [1]. The egg dissolves in the body if it is not fertilized. Menstruation, sometimes known as a "period," begins approximately 2 weeks thereafter. The sac that contained the egg should disintegrate when the egg is liberated during ovulation. If the egg fails to leave the sac in the ovary, or if the sac fills with fluid, a cyst forms [2].

Torsion of the ovary occurs when it twists over the ligaments that support it. When the fallopian tube twists with the ovary, this is referred to as adnexal torsion. Ovarian cysts are common during early pregnancy, however, these are usually harmless like other ovarian cysts. However, if the cysts continue to expand throughout your pregnancy, a few complications may arise. They may burst, twist, or possibly cause complications during birthing [3].

Indeed, ovarian cysts are seen in 0.2-2% of pregnancies and tend to be small and asymptomatic [4]. These cysts are usually discovered by chance during a prenatal ultrasound or when they develop symptoms. They are usually functional and improve by themselves before the end of the third trimester of pregnancy. The fact that they endure till the completion of the pregnancy lends credence to the cyst's organic nature [5].

This case report describes a gangrenous, twisted cyst in the ovary discovered in the first trimester of pregnancy.

## Case presentation

A 25-year-old multigravida came to the outpatient department with chief complaints of amenorrhea for 1 month. She complained of abdominal pain for 3 days which was sharp, dull aching, more on the right side, continuous, and non-radiating. It was unrelieved with medications.

She had conceived spontaneously with the obstetric formula G3P1L1A1, and the date of her last menstrual period was 4/7/23. She has a male child who is 18 months old and is alive and healthy.

**Figure 1:**
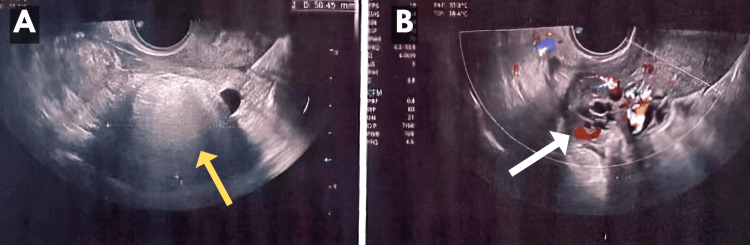
Ultrasonography findings A. Well-defined heterogeneously hyperechoic lesion of size 50x38 mm (yellow arrow); B. Right ovarian pedicle showing increased vascularity (white arrow)

The patient was cognizant and coherent during the examination, with a heart rate of 100 beats per minute, a blood pressure reading of 100/60 mmHg, a normal temperature, and normal cardiovascular and respiratory systems. Sonography was advised to the patient. Ultrasonography revealed uterus was mildly enlarged with an intrauterine gestational sac with a mean sac diameter of 6.5 mm, yolk sac was visible. The sonographic age was estimated to be 5 to 6 weeks. The sonography also showed a well-defined heterogeneous, hyperechoic lesion of size 50*38 mm seen in the right adnexa. The right ovarian pedicle appeared to be edematous and bulky and showed increased vascularity (Figure 1).

This was suggestive of a right ovarian cyst with torsion of the cyst.

**Figure 2:**
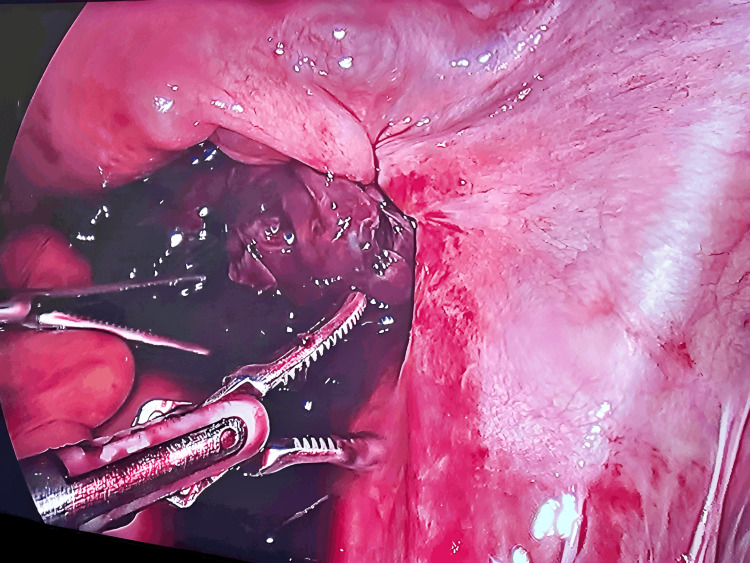
Laparoscopic image showing right ovarian gangrenous cyst

The patient was informed about her condition and given an emergency event, she was admitted for an emergency laparoscopic excision of cyst under general anesthesia. The patient was induced with intravenous propofol 100 mg and succinylcholine 75 mg. The procedure was started and on visualization of the right ovary, it was hemorrhagic, enlarged, and gangrenous (Figure 2).

**Figure 3:**
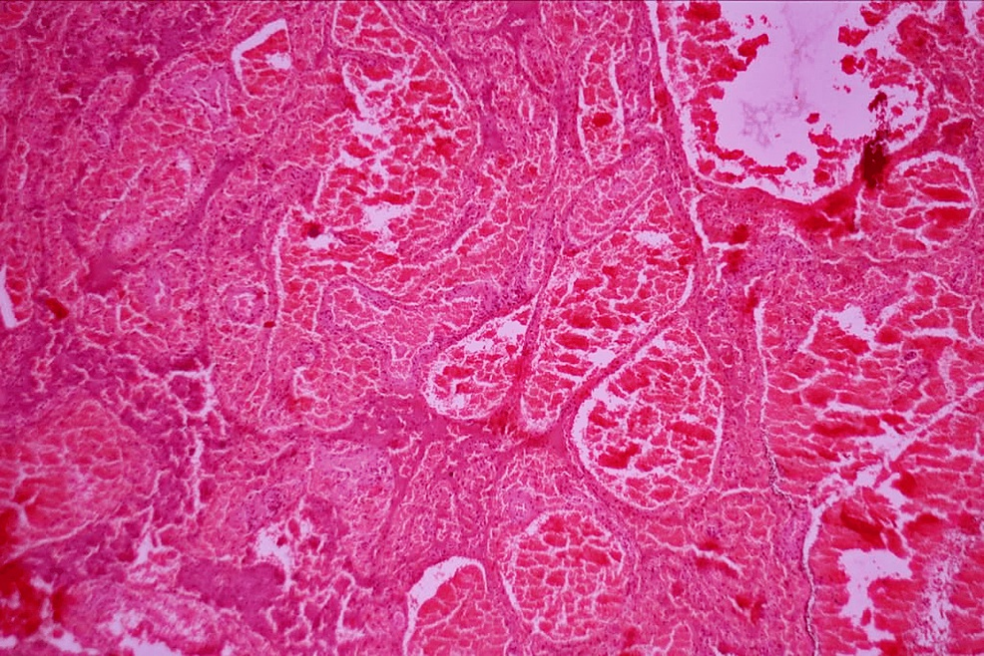
Histology slide showing cyst wall with marked hemorrhage and congestion

It was found that the cyst had twisted four times around its pedicle. Despite cautious detorsion, after 20 minutes there was no visible change in color or edema. After that, a right salpingo-oophorectomy was carried out and the sample was sent for biopsy. The patient had also opted for medical termination of pregnancy which was also done in the same sitting. The rest of the procedure was uneventful and the patient was shifted to the ward, hemodynamically and vitally stable. The patient was discharged two days post-op without any complications. The biopsy report showed evidence of a cyst wall with marked hemorrhage and congestion suggesting of benign hemorrhagic cyst (Figure 3).

## Discussion

Torsion of the ovaries during pregnancy is an uncommon complication that occurs more often in the third trimester of pregnancy and less frequently in the first. The symptoms are ambiguous and may be misdiagnosed as ureteral or renal, appendicitis, cholecystitis, or intestinal blockage [6]. The majority of ovarian cysts found during pregnancy are functional, such as corpus luteal cysts, follicular cysts, and hemorrhagic cysts. Among the numerous benign ovarian tumors discovered include serous cyst adenomas, cystic teratomas, para ovarian cysts, mucinous cyst adenomas, and endometriomas. Cancerous tumors are discovered seldom.

Ovarian cysts less than 6 cm in diameter which appear benign on ultrasonography are usually treated conservatively since they can dissolve spontaneously. Cysts greater than 10 cm are usually removed due to a greater likelihood of malignancy, torsion, or rupture. It is controversial whether cysts measuring 5 to 10 cm should be treated [7-9]. The treatment strategy is governed by the size of the cyst, the instruments available, and the doctor's level of experience. Many authors have advised not aspirating the cyst's contents since it raises the risk of complications such as infection, bleeding, peritoneal adhesion, and cyst rupture. Laparoscopic surgery, on the other hand, is possible. The trocars are placed and the contents of the cyst are aspirated before being removed [10].

Simple detorsion was performed using a laparoscopic method, however, the procedure was unsuccessful, and a salpingo-oophorectomy was required instead. Unfortunately, there was no way to preserve the ovary.

## Conclusions

Torsion of the ovaries is a gynecological emergency that may happen during pregnancy. In the first trimester of pregnancy, ovarian torsion is not all that prevalent. Typically, a diagnosis can be determined based on the typical clinical presentation and ultrasound evidence of a unilaterally enlarged adnexal mass. Surgery is the only available form of treatment, either through laparoscopy or laparotomy. To summarize, obstetricians should be aware of the risk of acute ovarian torsion in pregnant women. Both the mother and the unborn child will benefit from prompt surgical intervention.
